# Unlocking the role of silicon against biotic stress in plants

**DOI:** 10.3389/fpls.2024.1430804

**Published:** 2024-12-12

**Authors:** Krishan K. Verma, Xiu-Peng Song, Qiang Liang, Hai-Rong Huang, Rajan Bhatt, Lin Xu, Gan-Lin Chen, Yang-Rui Li

**Affiliations:** ^1^ Sugarcane Research Institute, Guangxi Academy of Agricultural Sciences/Key Laboratory of Sugarcane Biotechnology and Genetic Improvement, Ministry of Agriculture and Rural Affairs/Guangxi Key Laboratory of Sugarcane Genetic Improvement, Nanning, Guangxi, China; ^2^ Punjab Agricultural University (PAU)-Krishi Vigyan Kendra Amritsar, Punjab, India; ^3^ Guangxi Subtropical Crops Research Institute, Guangxi Academy of Agricultural Sciences, Nanning, Guangxi, China; ^4^ Guangxi Key Laboratory of Quality and Safety Control for Subtropical Fruits, Ministry of Agriculture and Rural Affairs, Nanning, Guangxi, China; ^5^ Key Laboratory of Quality and Safety Control for Subtropical Fruit and Vegetable, Ministry of Agriculture and Rural Affairs, Nanning, Guangxi, China; ^6^ School of Chemistry and Chemical Engineering, Guangxi Minzu University, Nanning, Guangxi, China

**Keywords:** pathogenic diseases, disease tolerance efficiency and management, crop productivity, plant nutrition, silicon

## Abstract

The requirement for agricultural crops continues to enhance with the continuous growth of the human population globally. Plant pathogenic diseases outbreaks are enhancing and threatening food security and safety for the vulnerable in different regions worldwide. Silicon (Si) is considered a non-essential element for plant growth. It regulates the biological functions, plant development and productivity, and balance the defense mechanism in response to fungal, bacterial and pest attacks. The optimum crop yield can be achieved by applying Si in agricultural systems through different methods to replace or minimize the use of synthetic fertilizers. This approach can be effective on crop production during limited resources, extreme climates, pests and diseases, and environmental pollution. Silicon can be applied as foliar spray, priming of seeds, soil water irrigation, soil amendment and soilless medium (hydroponic) to enhance plant performance and stress tolerance capacity during stress conditions. This article summarized the effective roles of Si and the ability to perform in agroecosystems for better crop production, food security and safety for sustainable agriculture in the future.

## Introduction

Silicon (Si) has a brittle crystalline structure with enormous application in biological sciences. Si finds a second position in the abundance of the earth’s crust (Verma et al., 2023a, b). Due to its affinity towards oxygen (Verma et al., 2019), Si forms two oxides, silica (SiO_2_) and silicon monoxide (SiO), as the SiO bond is unusually strong. The average Si content in normal soil is very high, i.e., 28% (weight basis). Soil carries silicon dioxide, silicate minerals, and alumino-silicates are not available to plants through their uptake process. The uptake and accumulation of Si depends on the variety of crop plants, soil properties, sources and concentration of Si *in planta*. Si content can vary from 0.1 to 10% (dry weight basis) near the detection limit (Coskun et al., 2019; Verma et al., 2020a, b; 2023a). Monosilicic acid is soluble in water and adsorbed by plant roots (Matichenkov and Calvert, 2002; Verma et al., 2020c; Paton, 2023).

Interestingly, the concentration of plant-accessible Si exceeds the phosphorus in the soil solution. Different factors, including pH, water status, temperature, and accompanying ions, influence Si availability in the rhizospheric soil (Bityutskii et al., 2019; Verma et al., 2020d, e). Specific soil types, such as extensively weathered acidic soil and calcareous paddy soil, may experience Si deficiency. Si can be detected in nearly all land-dwelling plant species but in different concentrations range. Some plant species have optimum Si accumulation capacity, while most have relatively low levels (Verma et al., 2020f; Hernandez-Apaolaza, 2022).

Plant diseases negatively impact plants’ growth, development and food grain quality. It is a severe problem for sustainable agriculture and food security. Unsuitable agricultural approaches are degrading the atmospheric environmental variables and facing population pressure due to less crop productivity as required (Verma et al., 2021a, b). Si was reported to enhance the defense system against biotic stresses occurring in the form of plant pathogens, such as fungi, insects, weeds, bacteria and viruses or animals, i.e., vertebrates and arthropod herbivores. The deposition of Si upregulated the abrasiveness of plant tissues and thus reduced palatability and digestibility for herbivores (Massey and Hartley, 2009; Luyckx et al., 2017). The physical strength of the leaf resulting from the accumulation of Si can afford mechanical protection and control the frequency and severity of the infection (Zhang et al., 2013; Ning et al., 2014; Song et al., 2021). The postulation of potential physical obstruction formation is based on the type of Si deposition in foliage, especially in the cell wall. Si in the plant cell wall and apoplast protects pathogen penetration (Luyckx et al., 2017; Gulzar et al., 2021). Recent demonstrations have shown that the biochemical mechanisms of Si compared to physical mechanisms play a significant role in enhancing plant tolerance efficiency during pathogenic diseases (Song et al., 2016, 2021).

The current global scenario presents humanity with an alarming level of hunger, primarily driven by rapid population pressure. Unfortunately, the limited availability of inadequate resources is incapable to fulfilling the demand of food (Verma et al., 2021c, d). The era of climate change is a significant obstacle to achieving sustainable agricultural productivity (Verma et al., 2024). However, biotic stress adds to the challenges faced by plant production systems worldwide, and a transition towards environmentally friendly approaches is necessary (Kumari et al., 2022; 2023; Verma et al., 2022a, b, c). Si offers a natural defense system against various insects and pathogens by strengthening plant tissues, inducing the production of defense compounds, and activating systemic defense responses. Si significantly reduce the reliance on synthetic pesticides, i.e., nematicides (Santos et al., 2022).

This review thoroughly discussed how Si mitigates biotic stress in plants. It provides technical and theoretical knowledge, and action of defense mechanisms on Si-based strategies to enhance plant resilience and productivity in adverse climatic conditions. It aims to understand how plants acquire and allocate Si in plant parts during biotic stress.

## Impact of biotic stress on plant development

Pathogenic diseases have significant implications for plant growth, development and crop protection. Fungal pathogens invade plant tissues, causing diseases, such as rust and powdery mildew, which cause wilting and reduce crop productivity and quality. Similarly, bacterial and viral pathogens release toxins that induce symptoms like wilting and rotting, disrupting plant cell processes, impeding nutrient absorption, and stunting plant growth (Muthu et al., 2022). Insects and pests damage plant tissues and downregulate plant growth and photosynthetic CO_2_ assimilation rate. These biotic stresses compromise photosynthesis, nutrient absorption, and plant defense mechanisms, making plants more vulnerable to secondary infections. Different plant species possess some specific action mechanisms for the uptake and accumulation of Si in other plant parts during stress conditions.

## Role of Si on insects, pests, and weeds infection

In natural ecosystems, plant communities coexist with plant-feeding insects. However, agricultural productivity is directly damaged in agricultural systems by attacks from pathogens, pests, and weeds (Al-Gaashani et al., 2023). The impact of pest attacks on plants is observed by their effect on yield, which refers to the amount of economically viable product per unit area. Insect pests pose a significant threat to crops for human and animal consumption, directly damaging plants. In agri-systems, direct losses caused by pathogens and weeds account for a significant portion of the reduction in global crop production (Sharma et al., 2017; El-Ramady et al., 2022). Wheat and cotton were particularly susceptible, with potential losses exceeding 80%. Rice is affected by more than 800 insect species, resulting in actual losses of nearly 40% worldwide. In India, yield loss (21-50%) was observed in the rice by insect pests attack (Fahad et al., 2019). In wheat, actual losses of more than 30% worldwide (Jasrotia et al., 2021). Substantial yield loss was also observed in sorghum by pest challenges, such as *Atherigona soccata Rondani* and stem borer (**Table 1**).

**Table 1:** Potential of Si against biotic stress mitigation strategies.

Crop	Pathogen	Response	Source
Pumpkin (*Cucurbita pepo* L. Howden)	*Podosphaera xanthii*	Reduce powdery mildew disease and increase crop productivity, soil nutritional efficiency and uptake and accumulation of silicon	Lepolu Torlon et al., 2016
Bitter gourd (*Momordica charantia* L.)	*Erysiphe* sp.	Suppress disease severity and upregulate enzymatic activities, i.e., POD, PPO and pathogenesis-related genes, chitinase and β-1,3-glucanase.	Ratnayake et al., 2016
Chili pepper (*Capsicum annuum* ‘Muria F_1_)	*Colletotrichum gloeosporioides*	No significant impacts on plant development and fruit/flowering quality. Cell wall-bound phenolic compounds and thickness of cuticle were increased by the application of Si.	Jayawardana et al., 2015
Melon (*Cucumis melo* L.)	*Acidovorax citrulli*	Improved plant nutritional status, and minimize the bacterial fruit blotch disease	Conceiaco et al., 2014; Ferreira et al., 2015
Tomato (*Solanum lycopersicum*)	*Ralstonia solanacearum*, *Tuba absoluta* and *Colletotrichum dematium*	Control disease index, and enhanced Si accumulation in roots, soil bacterial content and actinomycetes and downregulate fungi/soil bacterial ratio (ca. 54%). Changes the soil microorganisms and enzymatic activities. Upregulate gene expressions in salicylic acid pathway but downregulate jasmonic acid and ethylene expression genes. Suppress disease resistance capacity. Controls leaf miner due to toxic effect of *Tuta absoluta* during larval stage.	Wang et al., 2013; dos Santos et al., 2015; Chen et al., 2015; Somapala et al., 2015
Cucumber (*Cucumis sativus* L.)	*Meloidogyne incognita*	Significantly minimize root-knot nematode activity	Dugui-Es et al., 2010
Soybean (*Glycine max* L.)		Downregulate the silver leaf white fly population	Ferreira and Moraes, 2011
Arabidopsis (*Arabidopsis thaliana* L.)	*Erysiphe cichoracearum*	Suppress disease and balance mechanical resistance capacity	Ghanmi et al., 2004
Banana (*Musa* spp. cv. Maca)	*Fusarium oxysporum* f. sp. *cubense*	Si level enhanced in the roots and reduced disease symptoms upto 27%. Lignin deposited in the roots cortex. Resist phenylpropanoid pathway during disease infection with Si application.	Fortunato et al., 2014
Banana (*Musa acuminate* L.)	*Cyllindrocladium spathiphylli* and *Pseudocercospora fijiensis*	Reduced root necrosis disease and enhanced plant growth after Si-application during disease infection. The disease severity index (DSI) reduced during 21 and 35-days of pathogen inoculation.	Vermeire et al., 2011; Gbongue et al., 2019
Barley (*Hordeum vulgare* L.)	*Blumeria graminis* f. sp. *Hordei race* A6	Enhanced pathogen inoculation resistance efficiency.	Wiese et al., 2005
Bell Pepper, Sakata Hybrid Xpp 6115 (*Capsicum annuum* L.)	*Phytiophthora capsici*	Enhanced Si concentration uptake in roots but not in stems during Si application on disease infected plants. Disease and relative lesion extension were downregulated and plant drymass enhanced. Si reduced the disease severity and upgrade plant growth and development.	French-Monar et al., 2010
Carrot (*Daucus carota* L.)	*Pectobacterium carotovorum* pv. *carotovorum*	Enhanced plant development, photosynthetic pigments, dry weight during pathogenic inoculated plants with Si application.	Siddiqui et al., 2020
Coffee (*Coffea Arabica* L.)	*Hemileia vastatrix*	The more Si deposition on the plant leaves Disease severity reduced the application of Si on inoculation plants.	Carre-Missio et al., 2014
Melon (*Cucumis melo* L.)	*Podosphaera xanthii*	The disease curve was minimize (65% and 73%), infection efficiency, expansion rate of colony, colony area, conidial production during foliar and root irrigation of Si application.	Dallagnol et al., 2012
Cotton (*Gossypium hirsutum iL.*)	*Fusarium oxysporum* f.sp. *vasinfectum*	Significant phenolic compounds were present in root during Si application followed by pathogenic inoculation. The lignin content in roots found higher than inoculated plants without Si. Si may affect cellular defense systems in cotton roots.	Whan et al., 2016
Asian Ginseng (*Panax ginseng* L.)	*Ilyonectria morspanacis*	Minimize disease severity, no direct effects against the pathogen. Decreased expression of *PgSWEET* leading to regulated sugar efflux into apoplast and increased resistance efficiency against applied pathogen.	Abbai et al., 2019
Oat (*Avena sativa* L.)	*Rhizoctonia solani* Kuhn	Physio-biochemical responses decreased during fungal inoculation with Si application. Si assists to protect the harmful effects caused by fungal inoculation. Disease index reduced when the fungus was applied with Si application.	Ahmad et al., 2023
Finger millet (*Eleusine coracana* Gaertn.)	*Sesamia inferens* Walker.	Si application induces the interactive action defense mechanism by upregulating the transcript level of silicon transporter genes (*EcLsi1, EcLsi2* and *EcLsi6*) and defense hormone regulating genes (*EcSAM, EcPAL* and *EcLOX*) during 72 hr of post infestation in stem and roots	Jadhao et al., 2020

Rapeseed-mustard crops damage from pests, like mustard aphids, leading to yield losses (35-73%) in India (Tyczewska et al., 2018). Leguminous crops, like chickpeas and pigeon peas are affected by various pests, which result in considerable yield losses. Forage leguminous plants such as berseem, alfalfa, and Persian clover encounter challenges from the pod borer, *H. armigera*, leading to substantial losses in seed yield. Cotton plants require the optimum amounts of insecticides compared to other crop plants. Insecticides account for a significant portion of the total expenditure in cotton production (15-42%). Cotton accounts for nearly 23% of the worldwide insecticide use (Shahrajabian et al., 2020). Various insect pests, including whiteflies, bollworms, aphids, and various sucking pests, cause yield losses (up to 82%). Bollworms have triggered yield losses before 20 years (Lashari et al., 2022).

Pest insects pose a significant damage to global food production. Much more research is available to support the idea that Si application on plants can boost resistance to insects, pests, and diseases, leading to increased crop yield (Song et al., 2021). Si deposits in monocots can act as a “mechanical barrier against insects,” and their active role in biological resistance is now acknowledged. Si is a promoter in triggering biological resistance by generating compounds like tannic and phenolic chemicals (Tayade et al., 2022). Application of Si exhibits resistance to stalk borer damage. It was demonstrated that applying Si to maize plants reduces larval survival of the borer. Increasing the silica content and lowered larva survival efficiency (Rajput et al., 2021). An association of notable significance was discovered between the resistance of maize to the subsequent generation of *Ostrinia nubilalis Hübnera* and the Si concentration present within the sheath and collar tissues. Si in the epidermis of plant leaves can dislodge young borer larvae, hindering their establishment in the stem. It is well known that Si increases plant tissue toughness, interfering with insect larval boring and feeding. High Si content in rice plants damages the mandibles of rice stem borer larvae (Juma et al., 2015; Cabrera-Ponce et al., 2019). The physical arrangement of Si along the sheath of leaves could cause varietal resistance to the insects. When plants attack, signaling cascades are triggered, regulating target genes by entering proteins into the nucleus. The soluble Si protects cucumbers against fungal diseases (Song et al., 2021; El-Ramady et al., 2022).

By developing physical barrier, Si deposition under leaf cuticles enhances plants’ resistance to insect pests. It makes the tissue rigid and abrasive, reducing palatability and digestibility for herbivores (Nikpay et al., 2023). Differences in epidermal Si deposition among cultivars contribute to variations in resistance. Leaf abrasiveness and digestibility are influenced by spine and phytolith morphology. Si upregulates the gene expression associated with defense systems and promotes the accumulation of defensive compounds. It improves pest resistance in wheat and cucumber, and upregulates the activities of defensive enzymes. In response to pathogen infection, Si-mediated defense includes forming papilla, developing callose, and accumulating phenolic compounds. Si hinders fungal ET production, preventing suppression of the innate immune system and enhancing resistance against brown spot disease in rice plants (Akhtar et al., 2018) (**Table 2**). The cell wall of fungi consists of the carbohydrates chitin and β-1,3-glucan, Si-induced chitinases, and β-1,3-glucanases enzymes can hydrolyze these compounds to oligosaccharides and, as a result, the plant’s defense responses are elicited (Cruz et al., 2013). Si-enhanced tolerance to fungal diseases, there is limited information is available on the Si and bacterial disease interaction in plants (Song et al., 2016).

**Table 2:** Plant pathogens and insects reported to be suppressed by Si application.

Type of pathogen and insect	Host	Pathogen	Source
Fungal	Arabidopsis (*Arabidopsis thaliana* L.)	*Erysiphe cichoracearum*	Fauteux et al., 2006; Vivancos et al., 2015
	Banana (*Musa* spp. Cv. Maca)	*Mycosphaerella fijiensis, Fusarium oxysporum f.* spp. *Cubense* and *Cylindrocladium spathiphylli*	Kablan et al., 2012; Fortunato et al., 2015
	Barley (*Hordeum vulgare* L.)	*Blumeria graminis*	Wiese et al., 2005
	Bean (*Phaseolus vulgaris* L.)	*Pseudocercospora griseola*	Rodrigues et al., 2010
	Pepper (*Capsicum annuum* L.)	*Phytophthora capsici*	French-Monar et al., 2010
	Bentgrass (*Agrostis stolonifera* L.)	*Sclerotinia homoeocarpa*	Zhang et al., 2006
	Bitter gourd (*Momordica charantia* L.)	*Erysiphe* spp.	Ratnayake et al., 2016
	Capsicum (*Capsicum annuum* L.)	*Colletotrichum gloeosporioides*	Jayawardana et al., 2016
	Wheat (*Triticum aestivum* L.)	*Pyrenophora tritici-repentis*	Dorneles et al., 2017; Pazdiora et al., 2018
	Soybean (*Glycine max* L.)	*P. sojae*	Guerin et al., 2014; Rasoolizadeh et al., 2018
Virus	Mango (*Mangifera indica* L.)	*P. syringae* pv. *syringae*	Gutierrez-Barranquero et al., 2012
	Tobacco (*Nicotiana tabacum* L.)	*Tobacco ringspot virus*	Zellner et al., 2011
Bacterial	Banana (*Musa* spp.)	*Xanthomonas campestris*	Mburu et al., 2016
	Cotton (*Gossypium* spp.)	*X. citri subsp. Malvacearum*	Oliveira et al., 2012
	Melon (*Cucumis melo* L.)	*Acidovorax citrulli*	Conceiaço et al., 2014
	Rice (*Oryza sativa* L.)	*X. oryzae* pv. *oryza*	Song et al., 2016
	Sweet pepper (*Capsicum annuum* L.)	*Ralstonia solanacearum*	Alves et al., 2015
	Tomato (*Solanum lycopersicum* L.)	*Pseudomonas syringae* and *Ralstonia solanacearum*	Andrade et al., 2013; Chen et al., 2014; Ghareeb et al., 2011; Jiang et al., 2019
	Wheat (*Triticum aestivum* L.)	*X. translucens*	Silva et al., 2010a
Nematode	Coffee (*Coffea arabica* L.)	*Meloidogyne exigua*	Silva et al., 2010b
	Rice (*Oryza sativa* L.)	*M. graminicola*	Zhan et al., 2018
Chewing	Sugarcane (*Saccharum officinarum* L.)	*Diatraea saccharalis, Eldana saccharina* and *E. saccharina*	Meyer and Keeping, 2001; Keeping and Meyer, 2002, 2006
	Alfalfa (*Medicago truncatula* L.)	*Beet armyworm*	Korth et al., 2006
	Rice (*Oryza sativa* L.)	*Chilo suppressalis* (Walker) (Lepidoptera: Crambidae)	Hou and Han, 2010
	Maize (*Zea mays* L.)	*Busseola fusca*	Juma et al., 2015
	Rice (*Oryza sativa* L. Susceptible)	*C. medinalis* Guenee	Han et al., 2015
	Cabbage (*Brassica oleracea* L.)	*Plutella xylostella*	Shoaib et al., 2018
	Rice (*Oryza sativa* L.)	*Scirpophaga incertulas* and *S. incertulas* (Walker)	Jeer et al., 2017; Han et al., 2018
	Soybean (*Glycine max* L.)	*Helicoverpa armigera*	Alves et al., 2018
Sucking	Wheat (*Triticum aestivum* L.)	*S. graminum* Rond.	Basagli et al., 2003; Goussain et al., 2005
	Maize (*Zea mays* L.)	*Rhopalosiphum maidis*	Moraes et al., 2005
	Cucumber (*Cucumis sativus* L.)	*(Gennadius* spp.) (Hemiptera: Aleyrodidae)	Correa et al., 2005
	Eggplant (*Solanum melongena* L.)	*Thysanoptera: Thripidae*	De Almeida et al., 2008
	Tomato (*Solanum lycopersicum* L.)	*Whitefly (Homoptera: Aleyrodidae)*	Inbar and Gerling, 2008
	Zinnia (*Zinnia elegans* Jacq.)	*Myzus persicae Sulzer*	Ranger et al., 2009
	Bean (*Phaseolus vulgaris* L.)	*Tetranychus urticae* Koh	Gatarayiha et al., 2010
	Rice (*Oryza sativa* L.)	*Cnaphalocrocis medinalis* Guenee	Ye et al., 2013
	Wheat (*Triticum aestivum* L.)	*Sitobion avenae* (F.) *(Hemiptera: Aphididae)*	Dias et al., 2014
	Maize (*Zea mays* L.)	*R. maidis*	Boer et al., 2019
	Rice (*Oryza sativa* L.)	*C. medinalis*	Liu et al., 2017
	Lime (*Citrus latifolia* L.)	*Diaphorina citri*	Ramirez-Godoy et al., 2018
Phloem Feeding	Grass species	*Sitobion avenae.*	Massey et al., 2006
	Rice (*Oryza sativa* L.)	*N. lugens*	Yang et al., 2018

Silicon nanoparticles (SiNPs) have shown efficacy as pesticides with the interactive application of commercial pesticides. SiNPs are assimilated into the cuticular lipids, resulting in the physical mortality of insects (Mittal et al., 2020). Applied SiNPs with garlic essential oil has successfully managed agricultural insect pests. SiNPs can act as nano-pesticides or nanocarriers, enhancing the effectiveness of commercial pesticides. SiNPs can lead to insect mortality through desiccation and damage to the digestive system. The effects of SiNPs on pests have primarily been studied in laboratory conditions, focusing on specific pests and applied concentration. Weeds exert a harmful influence on the crop yield loss. A nano-herbicide formulation has been developed to combat weeds. It enhances the accumulation of Si in plant tissues. Nanoparticles assist for better customization, facilitating improved penetration through the plant’s protective cuticle and targeted release of active ingredients. The loss in herbivore populations is notably noted in Si-accumulating and non-accumulating plants (Mathur and Srivastava, 2022; Sarraf et al., 2022; Verma et al., 2023a).

## Biochemical and molecular mechanisms of Si on plants to biotic stress

Si effectively mitigates biotic stresses in plants, serving as a key player in their adaptation and survival under unfavorable plant growth conditions. Si exerts its protective effects through various biochemical mechanisms, enabling plants to combat stresses effectively (Khan et al., 2024). It enhances the biochemical activities, i.e., peroxidase (POD), catalase (CAT), and superoxide dismutase (SOD). The generation of reactive oxygen species (ROS) and antioxidant metabolism have been linked with bacterial and fungal infection, and in response to damage from chewing and sucking insects (Debona et al., 2014; Yang et al., 2017; Frew et al., 2018). ROS played significant roles in different signaling pathways with plant hormones (Glazebrook, 2005; Torres, 2010). However, ROS can activate plant defense genes and the associated accumulation of defense metabolites, such as phytoalexins and allelochemicals (Thoma et al., 2003).

**Figure 1:**
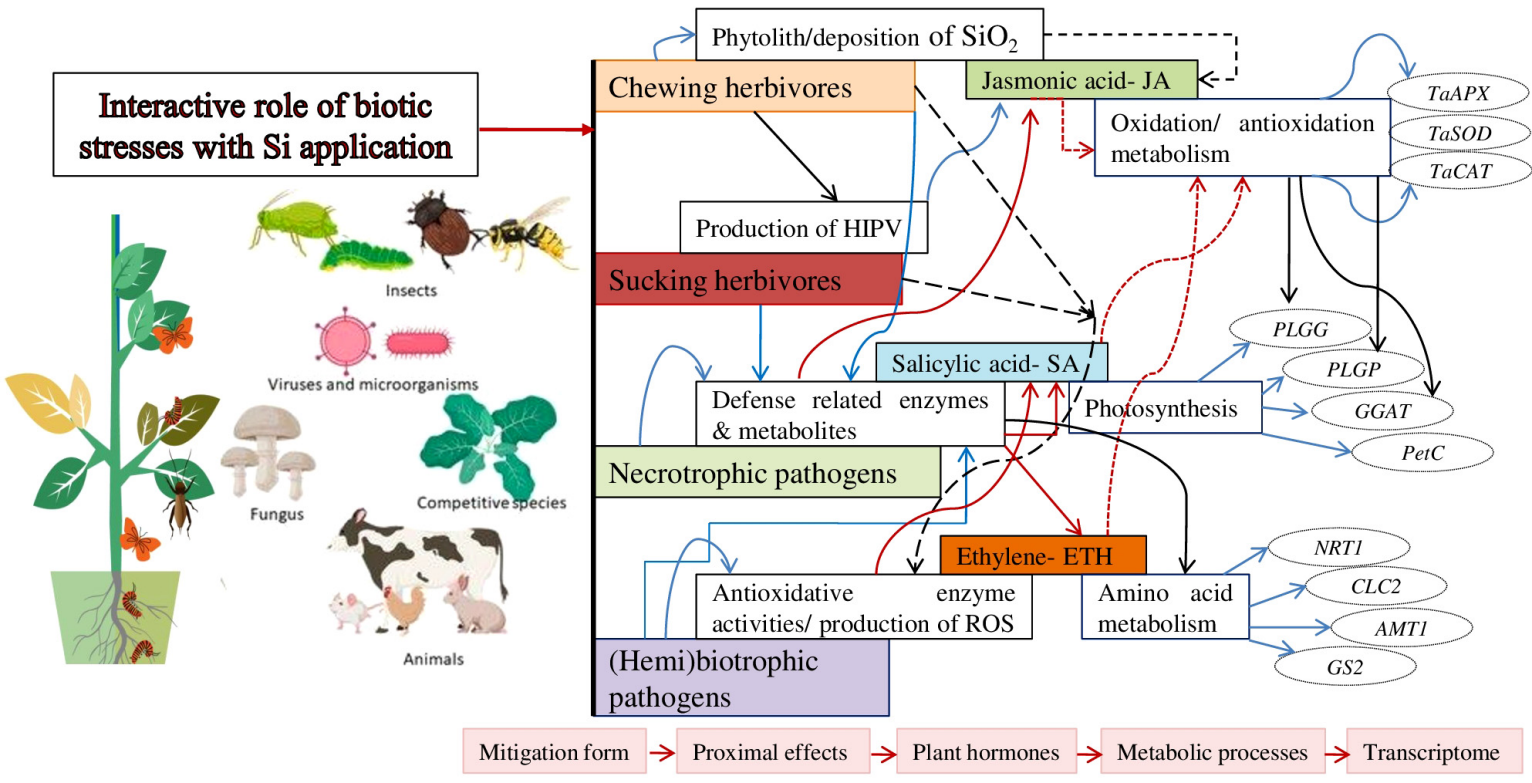
The outline of the major mitigation effects of Si on biotic stresses and some of the progressively more fundamental proximate and underlying phenomena associated with the mitigation of stress. The stress responsive genes associated with metabolic processes with variation in the response of transcription factors in response to Si. The essential Si transporter genes for the uptake of Si in plants are as *Lsi1, Lsi2, Lsi6* (Frew et al., 2018). Arrows shows linkages for which there is significant support and potential interactions.

Si plays a significant role during the uptake and translocation of nutritional elements. It enhances the absorption of essential nutrients from the rhizospheric soil, i.e., nitrogen, phosphorus, potassium, and calcium. It affects the action of nutrient transporters and balances the nutritional efficiency. It influences hormonal signaling pathways in plants, contributing to their ability to respond to stress. It involves the synthesis, transport, and signaling of phytohormones. Si induces the production of ABA, stress hormone implied in regulating stomatal closure and stress responses (Mukarram et al., 2022; Chen et al., 2024). Si also activates JA signaling pathways for plant defense mechanisms in response to biotic stresses (**Figure 1**).

Biochemical and molecular functions are also induced or reinforced by Si, allowing the plant to enhance stress tolerance efficiency and include defensive compounds, i.e., phenolics, phytoalexins and momilactons, but also activate the enzymatic defensive system, like polyphenol oxidase (PPO), lipoxygenase (LPO) and phenylalanine ammonia-lyase (PAL) (Remus-Borel et al., 2005; Rahman et al., 2015; Verma et al., 2021d). Applied Si can upregulate transcript levels corresponding to defensive-related genes during stressed conditions. Si also attracts predators or parasitoids to plants in response to herbivore attacks. However, soluble Si enhances herbivore-induced plant volatiles to upregulate or maintain predator attraction by pest-infected plants. The insect’s life cycle phenology is also downregulated in Si-applied plants, making it more prone to predation (Cai et al., 2008; Reynolds et al., 2016).

Si can control the stress-responsive gene expression, activating pathways that enhance plant tolerance efficiency. It upregulates stress-responsive transcription factors (SRTFs), heat shock proteins (HSPs), and pathogenesis-related proteins (Song et al., 2021; Mir et al., 2022). The modulation of gene expression, Si plays a significant role in triggering stress signaling pathways, facilitating the establishment of stress tolerance in plants (**Figure 1**). It promotes the accumulation of osmoprotectants, which are organic compounds that help to maintain cellular osmotic balance and protect against osmotic stress (Shomali et al., 2022, 2024). Subsequent solutes, such as sugars, proline, betaine, and glycine, accumulate in higher concentrations in Si-treated plants. These osmolytes act as osmoprotectants, maintaining cell turgor and stabilizing macromolecules, ultimately contributing to stress resilience (Song et al., 2021).

The application of transcriptomic strategies, i.e., microarrays alongside more targeted assays such as real-time quantitative PCR (qPCR) are critical in developing an understanding of how Si impacts the expression of plant genes. The pathogenic infection enhanced defense genes and reduced primary metabolism genes, but following the use of Si reduced genes were not as severely impacted, while they found limited information to suggest an impact of Si without pathogen stress (Fauteux et al., 2006). The application of Si nearly eradicated the effects of pathogen stress on the plant transcriptome. Some transcriptomic research work has been reported, limited research has assessed the impact of Si on enhanced plant resistance efficiency to insect herbivores. However, further studies on the interactions of Si with the transcriptome of a variety of crop plants varying in their Si uptake and accumulation ability, like accumulators, non-accumulators under different forms of insect herbivory, such as chewers, suckers should provide valuable insight into how Si changes plant gene expression in response to insect stressors (Chain et al., 2009; Bockhaven et al., 2015) (**Figure 1**).

## Mitigation of pathogenic bacteria, fungi, and viral diseases by Si application

Applying Si formulation open a new window in plant-pathogen interaction and management strategies. Si enhances resistance against diverse fungal pathogens in different plant-pathogen interactions. It effectively reduces plant diseases, such as black point in barley (*Alternaria* spp (Al-Sadi, 2021), leaf and glume blotch in wheat (*Septoria nodorum*), root rot in cucumber (*Pythium ultimum* and *Pythium aphanidermatum*) (Sun et al., 2022), damping off and stem rots in maize (*Pythium aphanidermatum*) (Haq et al., 2021), ascochyta blight in pea (*Mycosphaerella pinodes*), cercospora leaf spot in coffee (*Cercospora coffeicola*), blue mold decay and brown rot decay in cherry (*Penicillium expansum* and *Monilinia fructicola*), root rot in melon (*Pythium aphanidermatum*) (Sakr, 2018), wilt in potato (*Fusarium sulphureum*), black sigatoka in banana (*Mycosphaerella fijiensis*), and gray mold in strawberry (*Botrytis cinerea*). Foliar application of Si significantly reduces *Fusarium* crown and root rot in tomato plants. Si also reduces the intensity of hemibiotrophic fungal pathogens in different pathosystems, such as black spot in rose (*Diplocarpon rosae*), phytophthora blight in bell pepper (*Phytophthora capsici*), anthracnose in sorghum (*Colletotrichum sublineolum*), anthracnose in bean, and blast in wheat (*Pyricularia oryzae*). Si application reduces powdery mildew disease in wheat, cucumber, muskmelon, grape, arabidopsis, pearl millet, sugarcane, bean, strawberry, soybean, coffee, and rose, as well as powdery mildew in melon caused by *Podosphaera xanthii* (Farhat et al., 2018).

Si application upregulates plants’ efficiency for epiphytic and endophytic bacterial pathogens in mango plants where bacterial apical necrosis was reduced (Etesami et al., 2020; Verma et al., 2023b). Si treatments have also controlled bacterial wilt on tomato (Jiang et al., 2019), bacterial spot-on *Passiflora edulis*, bacterial streak on wheat, angular leaf spot on cotton, bacterial wilt on sweet pepper, and bacterial blight on rice. Applied calcium silicate in soil (1.41 g Si kg^−1^) defense against *Acidovorax citrulli* in melon plants (Bakhat et al., 2018). Different research groups’ demonstrations have confirmed the suppressive effects of Si application on viral pathogens. For instance, Si treatments have effectively reduced the incidence of *Cucumber mosaic virus* and *Papaya ring spot virus* on cucumbers. Applied Si in tobacco plants (0.1 mM) showed no systemic symptoms caused by the *Tobacco ring spot virus* than control plants, and higher Si rates slowed reduce the development of virus systemic symptoms. Si-mediated biotic stress tolerance by promoting various functions as shown in **Figure 1** and summarized in **Tables 1**, **2**.

## Conclusion and future prospects

Si can influence the ecophysiology and cellular metabolism of plants. It stimulates antioxidant mechanisms and photosynthetic apparatus, maintain nutritional balance, regulates nutrients’ uptake and accumulation, promotes the production of secondary metabolites, ROS, and toxic metal chelation, changes plant cell walls, and regulates stress resistance proteins. However, the most significant effect of Si is the reduction in the intensities and frequencies of different plant diseases caused by biotrophic, hemibiotrophic, and necrotrophic plant pathogens causing seed-borne, soilborne, and foliar diseases in a variety of crops of great economic importance. The plant responses during pathogen infection and pest attack at the physio-biochemical and molecular levels are remarkably similar when Si is taken up by the plant roots and translocated to shoots, indicating an active role played by this element in one or more plant defense signaling pathways. The regulatory functions of Si during stressed conditions discussed how Si tolerates stress efficiency. While Si is associated with various plant proteins, it is unclear which other transcription factors and signaling proteins interact with Si to enhance plant stress. It will be very interesting to discover the functional role of signaling pathways and interactions with phytohormones at the cellular level to understand better how plants react during biotic stress with Si application. However, upcoming research demonstrations should focus on deciphering the role of Si in crop plants at field trials rather than laboratory conditions. The CRISPR/Cas system should be explored to Si-encoding proteins to enhance the stress resistance capacity in response to pathogenic diseases in major crop plants.

## References

[B1] Abbai R. Kim Y. Mohanan P. Mathiyalagan R. Yang D. Rangaraj S.. (2019). Silicon confers protective effect against ginseng root rot by regulating sugar efflux into apoplast. Sci. Rep. 9, 1–10. doi: 10.1038/s41598-019-54678-x 31796825 PMC6890760

[B2] Ahmad F. Jabeen K. Iqbal S. Umar A. Ameen F. Gancarz M.. (2023). Influence of silicon nanoparticles on Avena sativa L. To alleviate the biotic stress of Rhizoctonia solani. Sci. Rep. 13, 1–15. doi: 10.1038/s41598-023-41699-w 37709782 PMC10502127

[B3] Akhtar N. Chandra R. Mazhar Z. (2018). Silicon based defence mechanism in plants. Trends Biosci. 11 (32), 3663–3674.

[B4] Al-Gaashani M. S. Samee N. A. Alnashwan R. Khayyat M. Muthanna M. S. A. (2023). Using a resnet50 with a kernel attention mechanism for rice disease diagnosis. Life 13, 1277. doi: 10.3390/life13061277 37374060 PMC10304950

[B5] Al-Sadi A. M. (2021). Bipolaris sorokiniana-induced black point, common root rot, and spot blotch diseases of wheat: A review. Front. Cell Infect. Microbiol. 11. doi: 10.3389/fcimb.2021.584899 PMC7991903 33777829

[B6] Alves A. L. Costa A. C. T. Mottin M. C. Pietrowiski V. Junior J. B. D. Nunes M. (2018). Evaluation of the insect pest population dynamics in common bean cultivars in relation to the foliar fertilisation with potassium silicate. J. Exp. Agric. Int. 27, 1–12. doi: 10.9734/jeai/2018/44031

[B7] Alves A. O. Santos M. M. B. Souza L. J. N. Souza E. B. Mariano R. L. R. (2015). Use of silicon for reducing the severity of bacterial wilt of sweet pepper. J. Plant Pathol. 97, 419–429. doi: 10.4454/JPP.V97I3.002

[B8] Andrade C. C. L. Resende R. S. Rodrigues F. A. Ferraz H. G. M. Moreira W. R. Oliveira J. R.. (2013). Silicon reduces bacterial speck development on tomato leaves. Trop. Plant Pathol. 38, 436–442. doi: 10.1590/S1982-56762013005000021

[B9] Bakhat H. F. Bibi N. Zia Z. Abbas S. Hammad H. M. Fahad S.. (2018). Silicon mitigates biotic stresses in crop plants: A review. J. Crop Prot. 104, 21–34. doi: 10.1016/j.cropro.2017.10.008

[B10] Basagli M. A. B. Moraes J. C. Carvalho G. A. Ecole C. C. Gonçalves-Gervásio R. de C. R. (2003). Efeito da aplicação de silicato de sódio na resistência de plantas de trigo ao pulgão-verde schizaphis graminum (Rond.) (Hemiptera: Aphididae). Neotrop. Entomol. 32, 659–663. doi: 10.1590/S1519-566X2003000400017

[B11] Bityutskii N. P. Yakkonen K. L. Petrova A. I. Lukina K. A. Shavarda A. L. (2019). Calcium carbonate reduces the effectiveness of soil-added monosilicic acid in cucumber plants. J. Soil Sci. Plant Nutr. 19, 660–670. doi: 10.1007/s42729-019-00066-3

[B12] Bockhaven J. V. Steppe K. Bauweraerts I. Kikuchi S. Asano T. Hofte M.. (2015). Primary metabolism plays a central role in moulding silicon-inducible brown spot resistance in rice. Mol. Plant Pathol. 16, 811–824. doi: 10.1111/mpp.12236 25583155 PMC6638399

[B13] Boer C. A. Sampaio M. V. Pereira H. S. (2019). Silicon-mediated and constitutive resistance to *Rhopalosiphum maidis* (Hemiptera: Aphididae) in corn hybrids. Bull. Entomol. Res. 109, 356–364. doi: 10.1017/S0007485318000585 30022743

[B14] Cabrera-Ponce J. L. Valencia-Lozano E. Trejo-Saavedra D. L. (2019). “Genetic modifications of corn,” in Corn, 3rd ed. (AACC International Press, Oxford). doi: 10.1016/B978-0-12-811971-6.00003-6

[B15] Cai K. Gao D. Luo S. Zeng R. Yang J. Zhu X. (2008). Physiological and cytological mechanisms of silicon-induced resistance in rice against blast disease. Physiol. Plant 134, 324–333. doi: 10.1111/j.1399-3054.2008.01140.x 18513376

[B16] Carre-Missio V. Rodrigues F. A. Schurt D. A. Resende R. S. Souza F. A. Rezende D. C.. (2014). Effect of foliar-applied potassium silicate on coffee leaf infection by Hemileia vastatrix. Ann. Appl. Biol. 164, 396–403. doi: 10.1111/aab.12109

[B17] Chain F. Côté-Beaulieu C. Belzile F. Menzies J. G. Bélanger R. R. (2009). A comprehensive transcriptomic analysis of the effect of silicon on wheat plants under control and pathogen stress conditions. Mol. Plant Microbe Int. 22, 1323–1330. doi: 10.1094/MPMI-22-11-1323 19810802

[B18] Chen D. Li C. Wu K. Xun G. Yuan S. Shen Q.. (2015). A phcA– marker-free mutant of Ralstonia solanacearum as potential biocontrol agent of tomato bacterial wilt. Biol. Contr. 80, 96–102. doi: 10.1016/j.biocontrol.2014.09.005

[B19] Chen J. Li Y. Zeng Z. Zhao X. Zhang Y. Li X.. (2024). Silicon induces ROS scavengers, hormone signalling, antifungal metabolites, and silicon deposition against brown stripe disease in sugarcane. Physiol. Plant 176, e14313. doi: 10.1111/ppl.14313 38666351

[B20] Chen Y. Liu M. Wang L. Lin W. Fan X. Cai K. (2014). Proteomic characterization of silicon-mediated resistance against *Ralstonia solanacearum* in tomato. Plant Soil 387, 425–440. doi: 10.1007/s11104-014-2293-4

[B21] Conceiaco C. S. Felix K. C. S. Mariano R. L. Medeiros E. V. Souza E. B. (2014). Combined effect of yeast and silicon on the control of bacterial fruit blotch in melon. Sci. Horticult. 174, 164–170. doi: 10.1016/j.scienta.2014.05.027

[B22] Correa R. S. B. Moraes J. C. Auad A. M. Carvalho G. A. (2005). Silício e acibenzolar-s-methyl como indutores de resistência em pepino, à mosca-branca *Bemisia tabaci* (Gennadius) (Hemiptera: Aleyrodidae) Biótipo B. Neotrop. Entomol. 34, 429–433. doi: 10.1590/S1519-566X2005000300011

[B23] Coskun D. Deshmukh R. Sonah H. Menzies J. G. Reynolds O. Ma J. F.. (2019). The controversies of silicon's role in plant biology. New Phytol. 221, 67–85. doi: 10.1111/nph.15343 30007071

[B24] Cruz M. Rodrigues F. A. Polanco L. R. Curvelo C. R. D. S. Nascimento K. J. T. Moreira M. A.. (2013). Inducers of resistance and silicon on the activity of defense enzymes in the soybean-*Phakopsora pachyrhizi* interaction. Bragantia 72, 162–172. doi: 10.1590/S0006-87052013005000025

[B25] Dallagnol L. J. Rodrigues F. A. Tanaka F. A. Amorim L. Camargo L. E. (2012). Effect of potassium silicate on epidemic components of powdery mildew on melon. Plant Pathol. 61, 323–330. doi: 10.1111/j.1365-3059.2011.02518.x

[B26] De Almeida G. D. Pratissoli D. Zanuncio J. C. Vicentini V. B. Holtz A. M. Serrão J. E. (2008). Calcium silicate and organic mineral fertilizer applications reduce phytophagy by *Thrips palmi* Karny (Thysanoptera: Thripidae) on eggplants (*Solanum melongena* L.). Interciencia 33, 835–838.

[B27] Debona D. Rodrigues F. A. Rios J. A. Nascimento K. J. T. Silva L. C. (2014). The effect of silicon on antioxidant metabolism of wheat leaves infected by *Pyricularia oryzae* . Plant Pathol. 6, 581–589. doi: 10.1111/ppa.2014.63.issue-3

[B28] Dias P. A. S. Sampaio M. V. Rodrigues M. P. Korndörfer A. P. Oliveira R. S. Ferreira S. E.. (2014). Induction of resistance by silicon in wheat plants to alate and apterous morphs of *Sitobion avenae* (Hemiptera: Aphididae). Environ. Entomol. 43, 949–956. doi: 10.1603/en13234 25182615

[B29] Dorneles K. R. Dallagnol L. J. Pazdiora P. C. Rodrigues F. A. Deuner S. (2017). Silicon potentiates biochemical defense responses of wheat against tan spot. Physiol. Mol. Plant Pathol. 97, 69–78. doi: 10.1016/j.pmpp.2017.01.001

[B30] dos Santos M. C. Junqueira M. R. de Sá V. M. Zanúncio J. C. Serrão J. E. (2015). Effect of silicon on the morphology of the midgut and mandible of tomato leaf miner *Tuta absoluta* (Lepidoptera: gelechiidae) larvae. Invertebr. Surviv. J. 12, 158–165.

[B31] Dugui-Es C. Pedroche N. Villanueva L. Galeng J. De D. W. (2010). Management of root knot nematode,Meloidogyne incognitain cucumber (*Cucumis sativus*) using silicon. Commun. Agric. Appl. Biol. Sci. 75, 497–505. doi: 10.3390/plants8060148 21539270

[B32] El-Ramady H. R. Verma K. K. Rajput V. D. Minkina T. Elbehiry F. Elbasiouny H. Y.. (2022). “Sources of silicon and nano-silicon in soil and plants,” in book: silicon and nano-silicon in environmental stress management and crop quality improvement: progress and prospects (United Kingdom: Academic Press, Elsevier). doi: 10.1016/B978-0-323-91225-9.00003-0

[B33] Etesami H. Jeong B. R. Rizwan M. (2020). “The use of silicon in stressed agriculture management,” in Metalloids in plants (Wiley Online Library, Hoboken). doi: 10.1002/9781119487210.ch19

[B34] Fahad S. Adnan M. Noor M. Arif M. Alam M. Khan I. A.. (2019). “Major constraints for global rice production,” in Advances in rice research for abiotic stress tolerance (Woodhead Publishing, Sawston). doi: 10.1016/B978-0-12-814332-2.00001-0

[B35] Farhat M. Haggag W. Thabet M. Mosa A. (2018). Efficacy of silicon and titanium nanoparticles biosynthesis by some antagonistic fungi and bacteria for controlling powdery mildew disease of wheat plants. Technol. 14, 661–674. Available at: https://www.thaiscience.info/Journals/Article/IJAT/10992391.pdf.

[B36] Fauteux F. Chain F. Belzile F. Menzies J. G. Bélanger R. R. (2006). The protective role of silicon in the Arabidopsis-powdery mildew pathosystem. Proc. Natl. Acad. Sci. U. S. A. 103, 17554–17559. doi: 10.1073/pnas.0606330103 17082308 PMC1859967

[B37] Ferreira H. A. do Nascimento C. W. A. Datnoff L. E. de Sousa Nunes G. H. Preston W. de Souza E. B.. (2015). Effects of silicon on resistance to bacterial fruit blotch and growth of melon. Crop Protect. 78, 277–283. doi: 10.1016/j.cropro.2015.09.025

[B38] Ferreira R. S. Moraes J. C. (2011). Silicon influence on resistance induction against *Bemisia tabaci* biotype B (Genn.) (Hemiptera: aleyrodidae) and on vegetative development in two soybean cultivars. Neotrop. Entomol. 40, 495–500. doi: 10.1590/S1519-566X2011000400014 21952968

[B39] Fortunato A. A. da Silva W. L. Rodrigues F. A. (2014). Phenylpropanoid pathway is potentiated by silicon in the roots of banana plants during the infection process of Fusarium oxysporum f. sp. cubense. Phytopathol. 104, 597–603. doi: 10.1094/PHYTO-07-13-0203-R 24350769

[B40] Fortunato A. A. Debona D. Bernardeli A. M. A. Rodrigues F. A. (2015). Defence-related enzymes in soybean resistance to target spot. J. Phytopathol. 163, 731–742. doi: 10.1111/jph.12370

[B41] French-Monar R. D. Rodrigues F. A. Korndörfer G. H. Datnoff L. E. (2010). Silicon suppresses phytophthora blight development on bell pepper. J. Phytopathol. 158, 554–560. doi: 10.1111/j.1439-0434.2009.01665.x

[B42] Frew A. Weston L. A. Reynolds O. L. Gurr G. M. (2018). The role of silicon in plant biology: a paradigm shift in research approach. Ann. Bot. 121, 1265–1273. doi: 10.1093/aob/mcy009 29438453 PMC6007437

[B43] Gatarayiha M. C. Laing M. D. Miller R. M. (2010). Combining applications of potassium silicate and Beauveria bassiana to four crops to control two spotted spider mite, *Tetranychus urticae* Koch. Int. J. Pest Manage. 56, 291–297. doi: 10.1080/09670874.2010.495794

[B44] Gbongue L.-R. Lalaymia I. Zeze A. Delvaux B. Declerck S. (2019). Increased Silicon Acquisition in Bananas Colonized by Rhizophagus irregularis MUCL 41833 reduces the incidence of *Pseudocercospora Fijiensis.* Front. Plant Sci. 9, 1977. doi: 10.3389/fpls.2018.01977 PMC6334260 30687370

[B45] Ghanmi D. McNally D. J. Benhamou N. Menzies J. G. Belanger R. R. (2004). Powdery mildew of Arabidopsis thaliana: A pathosystem for exploring the role of silicon in plant–microbe interactions. Physiol. Mol. Plant Pathol. 64, 189–199. doi: 10.1016/j.pmpp.2004.07.005

[B46] Ghareeb H. Bozsó Z. Ott P. G. Repenning C. Stahl F. Wydra K. (2011). Transcriptome of silicon-induced resistance against Ralstonia solanacearum in the silicon non-accumulator tomato implicates priming effect. Physiol. Mol. Plant Pathol. 75, 83–89. doi: 10.1016/j.pmpp.2010.11.004

[B47] Glazebrook J. (2005). Contrasting mechanisms of defense against biotrophic and necrotrophic pathogens. Ann. Rev. Phytopathol. 43, 205–227. doi: 10.1146/annurev.phyto.43.040204.135923 16078883

[B48] Goussain M. M. Prado E. Moraes J. C. (2005). Efeito do silício, aplicado em plantas de trigo, na biologia e comportamento alimentar do pulgão-verde *Schizaphis graminum* (Rond.) (Hemiptera: Aphididae). Neotrop. Entomol. 34, 807–813. doi: 10.1590/S1519-566X2005000500013

[B49] Guerin V. Lebreton A. Cogliati E. E. Hartley S. E. Belzile F. Menzies J. G.. (2014). A zoospore inoculation method with *Phytophthora Sojae* to assess the prophylactic role of silicon on soybean cultivars. Plant Dis. 98, 1632–1638. doi: 10.1094/PDIS-01-14-0102-RE 30703877

[B50] Gulzar N. Kamili A. N. Shah M. A. (2021). Silicon, the multifunctional element in reducing biotic and abiotic stress in plants. Int. Res. J. Plant Sci. 12, 1–9. doi: 10.14303/irjps.2021.20

[B51] Gutiérrez-Barranquero J. A. Arrebola E. Bonilla N. Sarmiento D. Cazorla F. M. De Vicente A. (2012). Environmentally friendly treatment alternatives to Bordeaux mixture for controlling bacterial apical necrosis (BAN) of mango. Plant Pathol. 61, 665–676. doi: 10.1111/j.1365-3059.2011.02559.x

[B52] Han Y. Lei W. Wen L. Hou M. (2015). Silicon-mediated resistance in a susceptible rice variety to the rice leaf folder, *Cnaphalocrocis medinalis* Guenée (Lepidoptera: Pyralidae). PloS One. 10 (4), e0120557. doi: 10.1371/journal.pone.0120557 25837635 PMC4383528

[B53] Han Y.-Q. Wen J.-H. Peng Z.-P. Zhang D.-Y. Hou M.-L. (2018). Effects of silicon amendment on the occurrence of rice insect pests and diseases in a field test. J. Integr. Agric. 17, 2172–2181. doi: 10.1016/S2095-3119(18)62035-0

[B54] Haq I. U. Khurshid A. Inayat R. Zhang K. Liu C. Ali S.. (2021). Silicon-based induced resistance in maize against fall armyworm [*Spodoptera frugiperda* (Lepidoptera: Noctuidae). PloS One 16, e0259749. doi: 10.1371/journal.pone.0259749 34752476 PMC8577731

[B55] Hernandez-Apaolaza L. (2022). Priming with silicon: a review of a promising tool to improve micronutrient deficiency symptoms. Front. Plant Sci. 13. doi: 10.3389/fpls.2022.840770 PMC8921768 35300007

[B56] Hou M. Han Y. (2010). Silicon-mediated rice plant resistance to the asiatic rice borer (Lepidoptera: Crambidae): effects of silicon amendment and rice varietal resistance. J. Econ. Entomol. 103, 1412–1419. doi: 10.1603/ec09341 20857756

[B57] Inbar M. Gerling D. (2008). Plant-mediated interactions between whiteflies, herbivores, and natural enemies. Annu. Rev. Entomol. 53, 431–448. doi: 10.1146/annurev.ento.53.032107.122456 17877454

[B58] Jadhao K. R. Bansal A. Rout G. R. (2020). Silicon amendment induces synergistic plant defense mechanism against pink stem borer (*Sesamia inferens* Walker.) in finger millet (*Eleusine coracana* Gaertn.). Sci. Rep. 10, 1–15. doi: 10.1038/s41598-020-61182-0 32144322 PMC7060215

[B59] Jasrotia P. Bhardwaj A. K. Katare S. Yadav J. Kashyap P. L. Kumar S.. (2021). Tillage intensity influences insect-pest and predator dynamics of wheat crop grown under different conservation agriculture practices in rice-wheat cropping system of indo-Gangetic plain. Agron. 11, 1087. doi: 10.3390/agronomy11061087

[B60] Jayawardana H. A. R. K. Weerahewa H. L. D. Saparamadu M. D. J. S. (2015). Enhanced resistance to anthracnose disease in chili pepper (*Capsicum annuum* L.) by amendment of the nutrient solution with silicon. J. Hortic. Sci. Biotechnol. 90, 557–562. doi: 10.1080/14620316.2015.11668714

[B61] Jayawardana H. A. R. K. Weerahewa H. L. D. Saparamadu M. D. J. S. (2016). The mechanisms underlying the anthracnose disease reduction by rice hull as a silicon source in capsicum (*Capsicum annuum* L.) grown in simplified hydroponics. Proc. Food Sci. 6, 147–150. doi: 10.1016/j.profoo.2016.02.035

[B62] Jeer M. Telugu U. M. Voleti S. R. Padmakumari A. P. (2017). Soil application of silicon reduces yellow stem borer, Scirpophaga incertulas (Walker) damage in rice. J. Appl. Entomol. 141, 189–201. doi: 10.1111/jen.12324

[B63] Jiang N. Fan X. Lin W. Wang G. Cai K. (2019). Transcriptome analysis reveals new insights into the bacterial wilt resistance mechanism mediated by silicon in tomato. Int. J. Mol. Sci. 20, 761. doi: 10.3390/ijms20030761 30754671 PMC6387441

[B64] Juma G. Ahuya P. Ong'amo G. Ru B. Magoma G. Silvain J.-F.. (2015). Influence of plant silicon in *Busseola fusca* (Lepidoptera: Noctuidae) larvae–Poaceae interactions. Bull. Entom. Res. 105, 253–258. doi: 10.1017/S000748531500005X 25633061

[B65] Kablan L. Lagauche A. Delvaux B. Legrève A. (2012). Silicon reduces black Sigatoka development in banana. Plant Dis. 96, 273–278. doi: 10.1094/PDIS-04-11-0274 30731798

[B66] Keeping M. G. Meyer J. H. (2002). Calcium silicate enhances resistance of sugarcane to the African stalk borer *Eldana saccharina* Walker (Lepidoptera: Pyralidae). Agric. For. Entomol. 4, 265–274. doi: 10.1046/j.1461-9563.2002.00150.x

[B67] Keeping M. G. Meyer J. H. (2006). Silicon-mediated resistance of sugarcane to Eldana saccharina Walker (Lepidoptera: Pyralidae): Effects of silicon source and cultivar. J. Appl. Entomol. 130, 410–420. doi: 10.1111/j.1439-0418.2006.01081.x

[B68] Khan S. R. Ahmad Z. Khan Z. Khan U. Asad M. Shah T. (2024). Synergistic effect of silicon and arbuscular mycorrhizal fungi reduces cadmium accumulation by regulating hormonal transduction and lignin accumulation in maize. Chemosphere 346, 140507. doi: 10.1016/j.chemosphere.2023.140507 38303379

[B69] Korth K. L. Doege S. J. Park S. H. Goggin F. L. Wang Q. Gomez S. K.. (2006). *Medicago truncatula* mutants demonstrate the role of plant calcium oxalate crystals as an effective defense against chewing insects. Plant Physiol. 141, 188–195. doi: 10.1104/pp.106.076737 16514014 PMC1459329

[B70] Kumari A. Dash M. Singh S. K. Jagadesh M. Mathpal B. Mishra P. K.. (2023). Soil microbes: a natural solution for mitigating the impact of climate change. Environ. Monit. Assess. 195, 1436. doi: 10.1007/s10661-023-11988-y 37940796

[B71] Kumari A. Lakshmi G. A. Krishna G. K. Patni B. Prakash S. Bhattacharyya M.. (2022). Climate change and its impact on crops: A comprehensive investigation for sustainable agriculture. Agronomy 12, 3008. doi: 10.3390/agronomy12123008

[B72] Lashari A. A. Korai S. K. Nizamani I. A. Qureshi K. H. Lodhi A. M. Korai A. K.. (2022). Monitoring of sucking pest on mustard crop through different colours sticky traps. Pak. J. Zool. 54, 801–808. doi: 10.17582/journal.pjz/20200427210427

[B73] Lepolu Torlon J. Heckman J. R. Simon J. E. Wyenandt C. A. (2016). Silicon soil amendments for suppressing powdery mildew on pumpkin. Sustainability 8, 293. doi: 10.3390/su8040293

[B74] Liu J. Zhu J. Zhang P. Han L. Reynolds O. L. Zeng R.. (2017). Silicon supplementation alters the composition of herbivore induced plant volatiles and enhances attraction of parasitoids to infested rice plants. Front. Plant Sci. 8. doi: 10.3389/fpls.2017.01265 PMC5515826 28769965

[B75] Luyckx M. Hausman J. F. Lutts S. Guerriero G. (2017). Silicon and plants: current knowledge and technological perspectives. Front. Plant Sci. 8. doi: 10.3389/fpls.2017.00411 PMC5362598 28386269

[B76] Massey F. P. Ennos A. R. Hartley S. E. (2006). Silica in grasses as a defence against insect herbivores: Contrasting effects on folivores and a phloem feeder. J. Anim. Ecol. 75, 595–603. doi: 10.1111/j.1365-2656.2006.01082.x 16638012

[B77] Massey F. P. Hartley S. E. (2009). Physical defences wear you down: progressive and irreversible impacts of silica on insect herbivores. J. Anim. Ecol. 78, 281–291. doi: 10.1111/j.1365-2656.2008.01472.x 18771503

[B78] Mathur P. G. J. Srivastava N. (2022). Silica nanoparticles as novel sustainable approach for plant growth and crop protection. Heliyon 8, e09908. doi: 10.1016/j.heliyon.2022.e09908 35847613 PMC9284391

[B79] Matichenkov V. V. Calvert D. V. (2002). Silicon as a beneficial element for sugarcane. J. Am. Soc Sug. Technol. 22, 21–30.

[B80] Mburu K. Oduor R. Mgutu A. Tripathi L. (2016). Silicon application enhances resistance to *xanthomonas* wilt disease in banana. Plant Pathol. 65, 807–818. doi: 10.1111/ppa.12468

[B81] Meyer J. H. Keeping M. G. (2001). Chapter 16 Past, present and future research of the role of silicon for sugarcane in southern Africa. Stud. Plant Sci. 8, 257–275. doi: 10.1016/S0928-3420(01)80020-3

[B82] Mir R. A. Bhat B. A. Yousuf H. Islam S. T. Raza A. Rizvi M. A.. (2022). Multidimensional role of silicon to activate resilient plant growth and to mitigate abiotic stress. Front. Plant Sci. 13. doi: 10.3389/fpls.2022.819658 PMC8984490 35401625

[B83] Mittal D. Kaur G. Singh P. Yadav K. Ali S. A. (2020). Nanoparticle-based sustainable agriculture and food science: Recent advances and future outlook. Front. Nanotechnol. 2. doi: 10.3389/fnano.2020.579954

[B84] Moraes J. C. Goussain M. M. Carvalho G. A. Costa R. R. (2005). Feeding non-preference of the corn leaf aphid *Rhopalosiphum maidis* (Fitc) (Hemiptera: Aphididae) to corn plants (*Zea mays* L.) treated with silicon. Ciec. e Agrotecnologia 29, 761–766. doi: 10.1590/s1413-70542005000400007

[B85] Mukarram M. Petrik P. Mushtaq Z. Khan M. M. A. Gulfishan M. Lux A. (2022). Silicon nanoparticles in higher plants: Uptake, action, stress tolerance, and crosstalk with phytohormones, antioxidants, and other signalling molecules. Environ. pollut. 310, 119855. doi: 10.1016/j.envpol.2022.119855 35940485

[B86] Muthu N. M. Ahmad N. Shivanand P. Metali F. (2022). The role of endophytes in combating fungal-and bacterial-induced stress in plants. Molecules 27, 6549. doi: 10.3390/molecules27196549 36235086 PMC9571366

[B87] Nikpay A. Tiwari A. K. Vejar-Cota G. Ziaee M. Wilson B. Srivastava S.. (2023). “Biotic stresses in sugarcane plants and its management,” in Agro-industrial perspectives on sugarcane production under environmental stress (Springer, Singapore). doi: 10.1007/978-981-19-3955-6_15

[B88] Ning D. Song A. Fan F. Li Z. Liang Y. (2014). Effects of slag-based silicon fertilizer on rice growth and brown-spot resistance. PloS One 9, e102681. doi: 10.1371/journal.pone.0102681 25036893 PMC4103847

[B89] Oliveira J. C. Albuquerque G. M. R. Mariano R. L. R. Gondim D. M. F. Oliveira J. T. A. Souza E. B. (2012). Reduction of the severity of angular leaf spot of cotton mediated by silicon. J. Plant Pathol. 94, 297–304.

[B90] Paton T. R. (2023). Soils: a new global view (London: CRC Press). doi: 10.1201/9781003420361

[B91] Pazdiora P. C. da Rosa Dorneles K. Forcelini C. A. Del Ponte E. M. Dallagnol L. J. (2018). Silicon suppresses tan spot development on wheat infected by *Pyrenophora tritici*-repentis. Eur. J. Plant Pathol. 150, 49–56. doi: 10.1007/s10658-017-1251-4

[B92] Rahman A. Wallis C. M. Uddin W. (2015). Silicon-induced systemic defense responses in perennial ryegrass against infection by *Magnaporthe oryzae* . Phytopathol. 105, 748–757. doi: 10.1094/PHYTO-12-14-0378-R 25738553

[B93] Rajput V. D. Minkina T. Feizi M. Kumari A. Khan M. Mandzhieva S.. (2021). Effects of silicon and silicon-based nanoparticles on rhizosphere microbiome, plant stress and growth. Biology 10, 791. doi: 10.3390/biology10080791 34440021 PMC8389584

[B94] Ramirez-Godoy A. Puentes-Pérez G. Restrepo-Díaz H. (2018). An evaluation of the use of calcium, potassium and silicon for the management of Diaphorina citri populations in Tahiti lime trees. Not. Bot. Horti Agrobot. Cluj-Napoca 46, 546–552. doi: 10.15835/nbha46211152

[B95] Ranger C. M. Singh A. P. Frantz J. M. Cañas L. Locke J. C. Reding M. E.. (2009). Influence of Silicon on Resistance of Zinnia elegans to Myzus persicae (Hemiptera: Aphididae). Environ. Entomol. 38, 129–136. doi: 10.1603/022.038.0116 19791606

[B96] Rasoolizadeh A. Labbé C. Sonah H. Deshmukh R. K. Belzile F. Menzies J. G.. (2018). Silicon protects soybean plants against *Phytophthora sojae* by interfering with effector-receptor expression. BMC Plant Biol. 18, 97. doi: 10.1186/s12870-018-1312-7 29848307 PMC5977513

[B97] Ratnayake R.M.R.N.K. Daundasekera W. A. M. Ariyarathne H. M. Ganehenege M. Y. U. (2016). Some biochemical defense responses enhanced by soluble silicon in bitter gourd-powdery mildew pathosystem. Australas. Plant Pathol. 45, 425–433. doi: 10.1007/s13313-016-0429-0

[B98] Remus-Borel W. Menzies J. G. Bélanger R. R. (2005). Silicon induces antifungal compounds in powdery mildew-infected wheat. Physiol. Mol. Plant Pathol. 66, 108–115. doi: 10.1016/j.pmpp.2005.05.006

[B99] Reynolds O. L. Padula M. P. Zeng R. Gurr G. M. (2016). Silicon: potential to promote direct and indirect effects on plant defence against arthropod pests in agriculture. Front. Plant Sci. 7. doi: 10.3389/fpls.2016.00744 PMC4904004 27379104

[B100] Rodrigues F.Á. Duarte H. S. S. Rezende D. C. Wordell Filho J. A. Korndorfer G. H. Zambolim L. (2010). Foliar spray of potassium silicate on the control of angular leaf spot on beans. J. Plant Nutr. 33, 2082–2093. doi: 10.1080/01904167.2010.519082

[B101] Sakr N. (2018). Silicon-enhanced resistance of plants to biotic stresses review article. Acta Phytopathol. Entomol. Hung. 53, 125–141. doi: 10.1556/038.53.2018.005

[B102] Santos L. B. de Souza Junior J. P. de Mello Prado R. Junior R. F. de Souza V. F. dos Santos Sarah M. M.. (2022). Silicon allows halving Cadusafos dose to control *Meloidogyne incognita* and increase cotton development. Silicon 14, 3809–3816. doi: 10.1007/s12633-021-01126-z

[B103] Sarraf M. Vishwakarma K. Kumar V. Arif N. Das S. Johnson R.. (2022). Metal/metalloid-based nanomaterials for plant abiotic stress tolerance: An overview of the mechanisms. Plants 11, 316. doi: 10.3390/plants11030316 35161297 PMC8839771

[B104] Shahrajabian M. H. Sun W. Cheng Q. (2020). Considering white gold, cotton, for its fiber, seed oil, traditional and modern health benefits. J. Biol. Environ. Sci. 14, 25–39.

[B105] Sharma S. Kooner R. Arora R. (2017). “Insect pests and crop losses,” in Breeding insect resistant crops for sustainable agriculture (Springer, Singapore). doi: 10.1007/978-981-10-6056-4_2

[B106] Shoaib A. Elabasy A. Waqas M. Lin L. Cheng X. Zhang Q.. (2018). Entomotoxic effect of silicon dioxide nanoparticles on *Plutella xylostella* (L.) (Lepidoptera: Plutellidae) under laboratory conditions. Toxicol. Environ. Chem. 100, 80–91. doi: 10.1080/02772248.2017.1387786

[B107] Shomali A. Das S. Arif N. Sarraf M. Zahra N. Yadav V.. (2022). Diverse physiological roles of flavonoids in plant environmental stress responses and tolerance. Plants 11, 3158. doi: 10.3390/plants11223158 36432887 PMC9699315

[B108] Shomali A. Das S. Sarraf M. Johnson R. Janeeshma E. Kumar V.. (2024). Modulation of plant photosynthetic processes during metal and metalloid stress, and strategies for manipulating photosynthesis-related traits. Plant Physiol. Biochem. 206, 108211. doi: 10.1016/j.plaphy.2023.108211 38029618

[B109] Siddiqui Z. A. Hashmi A. Khan M. R. Parveen A. (2020). Management of bacteria *Pectobacterium carotovorum, Xanthomonas campestris* pv. carotae, and fungi *Rhizoctonia solani, Fusarium solani* and *Alternaria dauci* with silicon dioxide nanoparticles on carrot. Int. J. Vegetable Sci. 26, 547–557. doi: 10.1080/19315260.2019.1675843

[B110] Silva R. V. Oliveira R. D. L. Nascimento K. J. T. Rodrigues F. A. (2010b). Biochemical responses of coffee resistance against Meloidogyne exigua mediated by silicon. Plant Pathol. 59, 586–593. doi: 10.1111/j.1365-3059.2009.02228.x

[B111] Silva I. T. Rodrigues F.Á. Oliveira J. R. Pereira S. C. Andrade C. C. L. Silveira P. R.. (2010a). Wheat resistance to bacterial leaf streak mediated by Silicon. J. Phytopathol. 158, 253–262. doi: 10.1111/j.1439-0434.2009.01610.x

[B112] Somapala K. Weerahewa H. L. D. Thrikawala S. (2015). Silicon rich rice hull amended soil enhances anthracnose resistance in tomato. Proc. Food Sci. 6, 190–193. doi: 10.1016/j.profoo.2016.02.046

[B113] Song X. P. Verma K. K. Tian D. D. Zhang X. Q. Liang Y. J. Huang X.. (2021). Exploration of silicon functions to integrate with biotic stress tolerance and crop improvement. Biol. Res. 54, 19. doi: 10.1186/s40659-021-00344-4 34238380 PMC8265040

[B114] Song A. Xue G. Cui P. Fan F. Liu H. Yin C.. (2016). The role of silicon in enhancing resistance to bacterial blight of hydroponic- and soil-cultured rice. Sci. Rep. 6 (1), 1–13. doi: 10.1038/srep24640 PMC4835757 27091552

[B115] Sun S. Yang Z. Song Z. Wang N. Guo N. Niu J.. (2022). Silicon enhances plant resistance to Fusarium wilt by promoting antioxidant potential and photosynthetic capacity in cucumber (*Cucumis sativus* L.). Front. Plant Sci. 13. doi: 10.3389/fpls.2022.1011859 PMC9608603 36311065

[B116] Tayade R. Ghimire A. Khan W. Lay L. Attipoe J. Q. Kim Y. (2022). Silicon as a smart fertilizer for sustainability and crop improvement. Biomol. 12, 1027. doi: 10.3390/biom12081027 PMC9332292 35892337

[B117] Thoma I. Loeffler C. Sinha A. K. Gupta M. Krischke M. Steffan B.. (2003). Cyclopentenone isoprostanes induced by reactive oxygen species trigger defense gene activation and phytoalexin accumulation in plants. Plant J. 34, 363–375. doi: 10.1046/j.1365-313X.2003.01730.x 12713542

[B118] Torres M. A. (2010). ROS in biotic interactions. Physiol. Plant 138, 414–429. doi: 10.1111/j.1399-3054.2009.01326.x 20002601

[B119] Tyczewska A. Woźniak E. Gracz J. Kuczyński J. Twardowski T. (2018). Towards food security: current state and future prospects of agrobiotechnology. Trends Biotechnol. 36, 1219–1229. doi: 10.1016/j.tibtech.2018.07.008 30262405

[B120] Verma K. K. Anas M. Chen Z. Rajput V. D. Malviya M. K. Verma C. L.. (2020a). Silicon supply improves leaf gas exchange, antioxidant defense system and growth in sugarcane responsive to water limitation. Plants 9, 1032. doi: 10.3390/plants9081032 32823963 PMC7464948

[B121] Verma K. K. Li D. M. Singh M. Rajput V. D. Malviya M. K. Minkina T.. (2020b). Interactive role of silicon and plant-rhizobacteria mitigating abiotic stresses: A new approach for sustainable agriculture and climate change. Plants 9, 1055. doi: 10.3390/plants9091055 32824916 PMC7569970

[B122] Verma K. K. Liu X.-H. Wu K.-C. Singh R. K. Song Q. Q. Malviya M. K.. (2020f). The impact of silicon on photosynthetic and biochemical responses of sugarcane under different soil moisture levels. Silicon 12, 1355–1367. doi: 10.1007/s12633-019-00228-z

[B123] Verma K. K. Singh P. Song X.-P. Malviya M. K. Singh R. K. Chen G.-L.. (2020c). Mitigating climate change for sugarcane improvement: role of silicon in alleviating abiotic stresses. Sugar Tech. 22, 741–749. doi: 10.1007/s12355-020-00831-0

[B124] Verma K. K. Singh R. K. Song Q. Q. Singh P. Zhang B.-Q. Song X.-P.. (2019). Silicon alleviates drought stress of sugarcane plants by improving antioxidant responses. Biomed. J. Sci. Tech. Res. 17 (1), 2019. doi: 10.26717/BJSTR.2019.17.002957

[B125] Verma K. K. Song X. P. Budeguer F. Nikpay A. Enrique R. Singh M.. (2022a). Genetic engineering: an efficient approach to mitigating biotic and abiotic stresses in sugarcane cultivation. Plant Signaling Behav. 17, e2108253. doi: 10.1080/15592324.2022.2108253 PMC9377231 35959678

[B126] Verma K. K. Song X. P. Joshi A. Tian D. D. Rajput V. D. Singh M.. (2022c). Recent trends in nano-fertilizer for advancing sustainable agriculture under the era of climate change to ensure food security for future generations. Nanomaterials 12, 173. doi: 10.3390/nano12010173 35010126 PMC8746782

[B127] Verma K. K. Song P. Kumari A. Jagadesh M. Singh S. K. Bhatt R.. (2024). Climate change adaptation: Challenges for agricultural sustainability. Plant Cell Environ. doi: 10.1111/pce.15078 39136256

[B128] Verma K. K. Song X. P. Li D. M. Singh M. Wu J. M. Singh R. K.. (2022b). Silicon and soil microorganisms improves rhizospheric soil health with bacterial community, plant growth, performance and yield. Plant Signaling Behav. 17, e2104004. doi: 10.1080/15592324.2022.2104004 PMC9364706 35943127

[B129] Verma K. K. Song X. P. Lin B. Guo D. J. Singh M. Rajput V. D.. (2021a). Silicon induced drought tolerance in crop plants: physiological adaptation strategies. Silicon 14 (6), 2473–2487. doi: 10.1007/s12633-021-01071-x

[B130] Verma K. K. Song X. P. Singh M. Tian D. D. Rajput V. D. Minkina T.. (2023a). Association of silicon and soil microorganisms induces stress mitigation, increasing plant productivity. Benefits Silicon Nutr. Plants pp, 299–328. doi: 10.1007/978-3-031-26673-7_17

[B131] Verma K. K. Song X. Tian D. D. Guo D. Chen Z. Zhong C.. (2021d). Influence of silicon on biocontrol strategies to manage biotic stress for crop protection, performance, and improvement. Plants 10, 2163. doi: 10.3390/plants10102163 34685972 PMC8537781

[B132] Verma K. K. Song X. P. Tian D. D. Singh M. Verma C. L. Rajput V. D.. (2021b). Investigation of defensive role of silicon during drought stress induced by irrigation capacity in sugarcane: physiological and biochemical characteristics. ACS Omega 6, 19811–19821. doi: 10.1021/acsomega.1c02519 34368568 PMC8340432

[B133] Verma K. K. Song X. Verma C. L. Huang H. Singh M. Xu L.. (2023b). Mathematical modeling of climate and fluoride effects on sugarcane photosynthesis with silicon nanoparticles. Plant Physiol. Biochem. 204, 108089. doi: 10.1016/j.plaphy.2023.108089 37852069

[B134] Verma K. K. Song X. P. Zeng Y. Guo D. J. Singh M. Rajput V. D.. (2021c). Foliar application of silicon boosts growth, photosynthetic leaf gas exchange, antioxidative response and resistance to limited water irrigation in sugarcane (*Saccharum officinarum* L.). Plant Physiol. Biochem. 166, 582–592. doi: 10.1016/j.plaphy.2021.06.032 34175813

[B135] Verma K. K. Song X. P. Zeng Y. Li D. M. Guo D. J. Rajput V. D.. (2020d). Characteristics and correlation of leaf stomata and its relationship with photosynthesis on. Saccharum under different irrigation silicon application. ACS Omega 5, 24145–24153. doi: 10.1021/acsomega.0c03820 PMC7513552 32984737

[B136] Verma K. K. Wu K. C. Verma C. L. Li D. M. Malviya M. K. Singh R. K.. (2020e). Developing mathematical model for diurnal dynamics on photosynthetic responses in sugarcane responsive to different irrigation and silicon application. PeerJ 8, e10154. doi: 10.7717/peerj.10154 33194396 PMC7597626

[B137] Vermeire M. L. Kablan L. Dorel M. Delvaux B. Risède J. M. Legrève A. (2011). Protective role of silicon in the banana-Cylindrocladium spathiphylli pathosystem. Eur. J. Plant Pathol. 131, 621–630. doi: 10.1007/s10658-011-9835-x

[B138] Vivancos J. Labbe C. Menzies J. G. Belanger R. R. (2015). Silicon-mediated resistance of Arabidopsis against powdery mildew involves mechanisms other than the salicylic acid (SA)- dependent defence pathway. Mol. Plant Pathol. 16, 572–582. doi: 10.1111/mpp.12213 25346281 PMC6638373

[B139] Wang L. Cai K. Chen Y. Wang G. (2013). Silicon-mediated tomato resistance against Ralstonia solanacearumis associated with modification of soil microbial community structure and activity. Biol. Trace Elem. Res. 152, 275–283. doi: 10.1007/s12011-013-9611-1 23371799

[B140] Whan J. A. Dann E. K. Aitken E. A. (2016). Effects of silicon treatment and inoculation with *Fusarium oxysporum* f. sp. vasinfectum on cellular defences in root tissues of two cotton cultivars. Ann. Bot. 118, 219–226. doi: 10.1093/aob/mcw095 27288509 PMC4970361

[B141] Wiese J. Wiese H. Schwartz J. Schubert S. (2005). Osmotic stress and silicon act additively in enhancing pathogen resistance in barley against barley powdery mildew. J. Plant Nutr. Soil Sci. 168, 269–274. doi: 10.1002/jpln.200420490

[B142] Yang L. Han Y. Li P. Li F. Ali S. Hou M. (2017). Silicon amendment is involved in the induction of plant defense responses to a phloem feeder. Sci. Rep. 7, 4232. doi: 10.1038/s41598-017-04571-2 28652621 PMC5484686

[B143] Yang L. Li P. Li F. Ali S. Sun X. Hou M. (2018). Silicon amendment to rice plants contributes to reduced feeding in a phloem-sucking insect through modulation of callose deposition. Ecol. Evol. 8, 631–637. doi: 10.1002/ece3.3653 29321899 PMC5756854

[B144] Ye M. Song Y. Long J. Wang R. Baerson S. R. Pan Z.. (2013). Priming of jasmonate-mediated antiherbivore defense responses in rice by silicon. Proc. Natl. Acad. Sci. U.S.A. 110, E3631–E3639. doi: 10.1073/pnas.1305848110 24003150 PMC3780902

[B145] Zellner W. Frantz J. Leisner S. (2011). Silicon delays Tobacco ringspot virus systemic symptoms in Nicotiana tabacum. J. Plant Physiol. 168, 1866–1869. doi: 10.1016/j.jplph.2011.04.002 21696845

[B146] Zhan L. P. Peng D. L. Wang X. L. Kong L. A. Peng H. Liu S. M.. (2018). Priming effect of root-applied silicon on the enhancement of induced resistance to the root-knot nematode *Meloidogyne graminicola* in rice. BMC Plant Biol. 18, 50. doi: 10.1186/s12870-018-1266-9 29580214 PMC5870084

[B147] Zhang Q. Fry J. Lowe K. Tisserat N. (2006). Evaluation of calcium silicate for brown patch and dollar spot suppression on turfgrasses. Crop Sci. 46, 1635–1643. doi: 10.2135/cropsci2005.04-0002

[B148] Zhang C. Wang L. Zhang W. Zhang F. (2013). Do lignification and silicification of the cell wall precede silicon deposition in the silica cell of the rice (*Oryza sativa* L.) leaf epidermis? Plant Soil 372, 137–149. doi: 10.1007/s11104-013-1723-z

